# Supplementary figures and images for: Thermography analysis as a tool for assessing thermal asymmetries and temperature changes after therapy in patients with stroke: a pilot study
Source: PeerJ. 2025 Aug 28;13:e19843. doi: 10.7717/peerj.19843 (PMC12399083; doi:10.7717/peerj.19843)

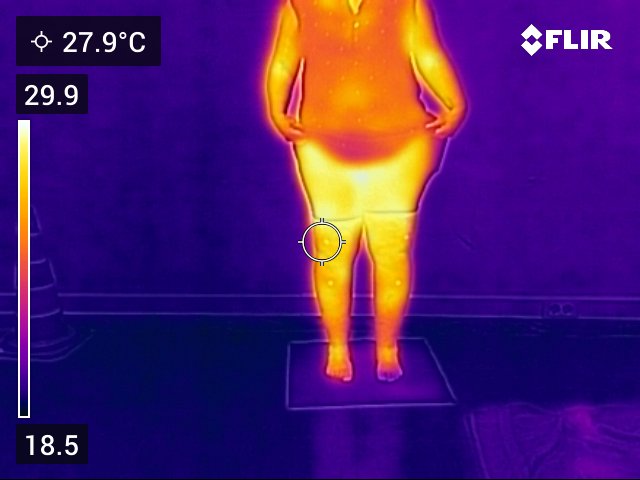

Supplement: Supplemental Information 2 [file peerj-13-19843-s002.zip › Fotos termografía/1_POST_Anterior.jpg]

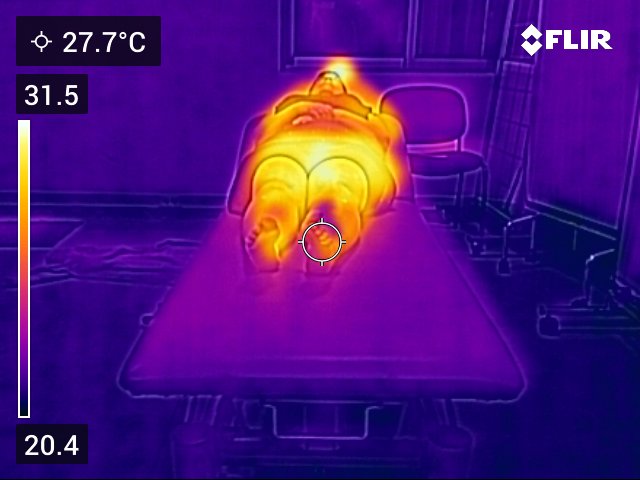

Supplement: Supplemental Information 2 [file peerj-13-19843-s002.zip › Fotos termografía/1_POST_Pies.jpg]

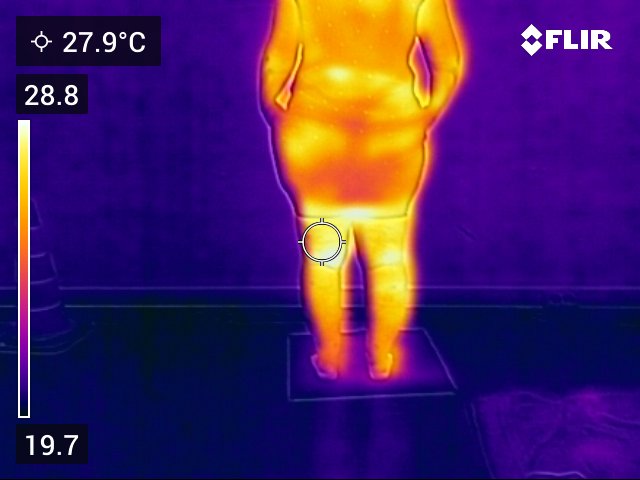

Supplement: Supplemental Information 2 [file peerj-13-19843-s002.zip › Fotos termografía/1_POST_Posterior.jpg]

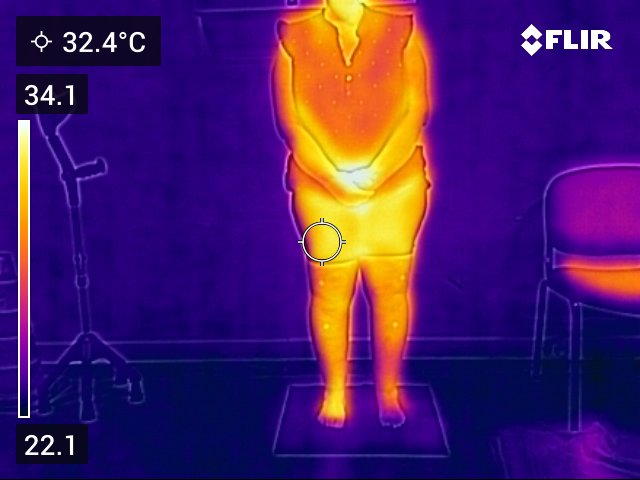

Supplement: Supplemental Information 2 [file peerj-13-19843-s002.zip › Fotos termografía/1_PRE_Anterior.jpg]

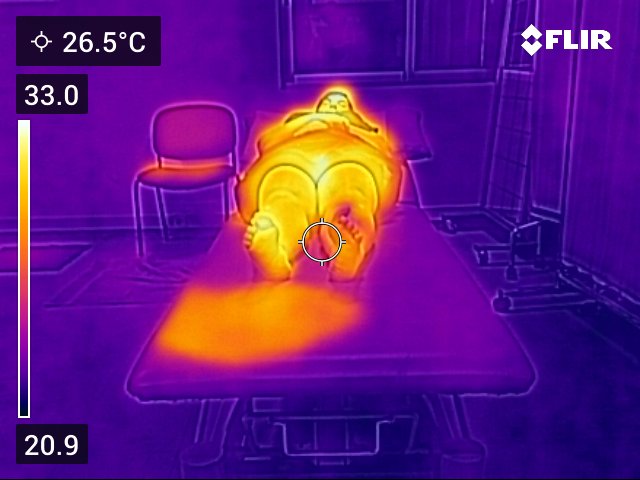

Supplement: Supplemental Information 2 [file peerj-13-19843-s002.zip › Fotos termografía/1_PRE_Pies.jpg]

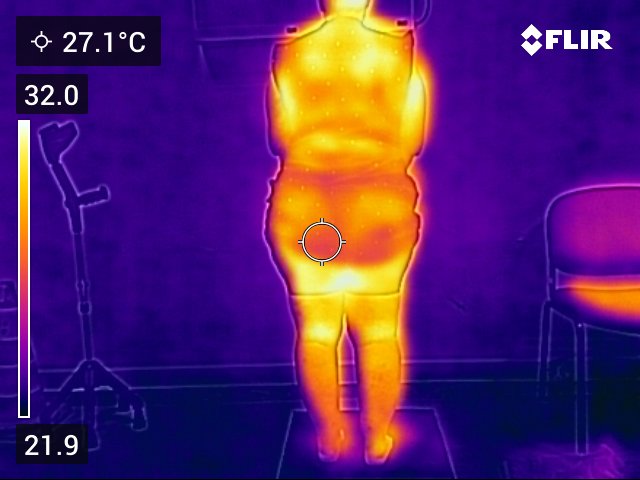

Supplement: Supplemental Information 2 [file peerj-13-19843-s002.zip › Fotos termografía/1_PRE_Posterior.jpg]

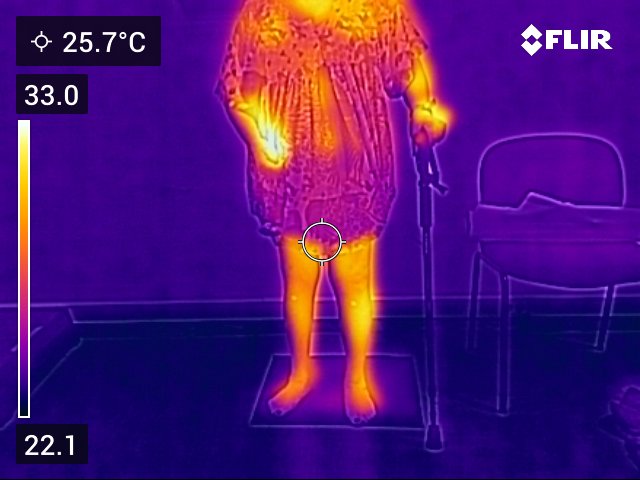

Supplement: Supplemental Information 2 [file peerj-13-19843-s002.zip › Fotos termografía/10_POST_Anterior.jpg]

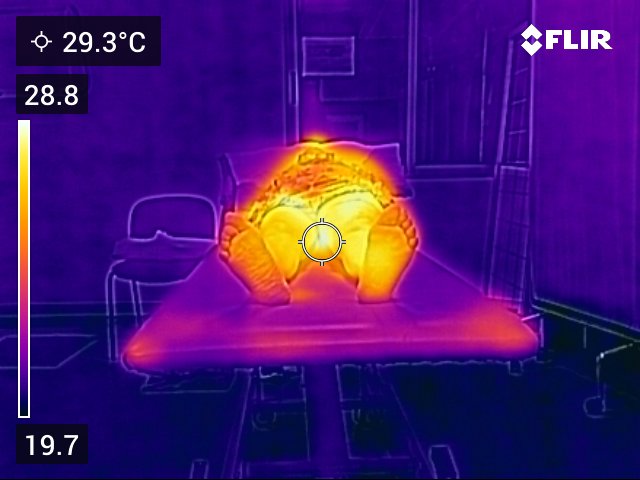

Supplement: Supplemental Information 2 [file peerj-13-19843-s002.zip › Fotos termografía/10_POST_Pies.jpg]

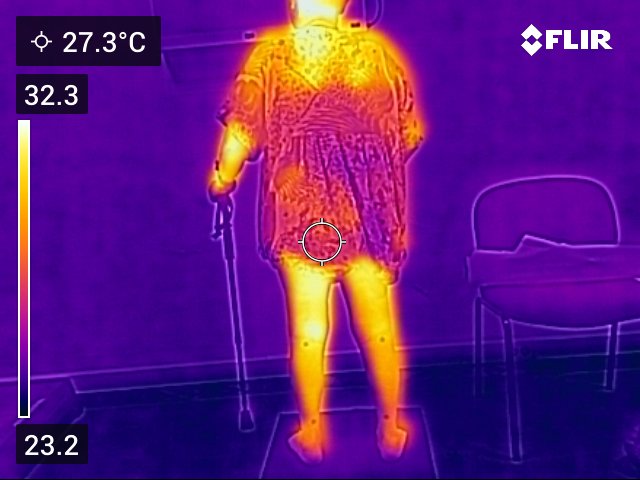

Supplement: Supplemental Information 2 [file peerj-13-19843-s002.zip › Fotos termografía/10_POST_Posterior.jpg]

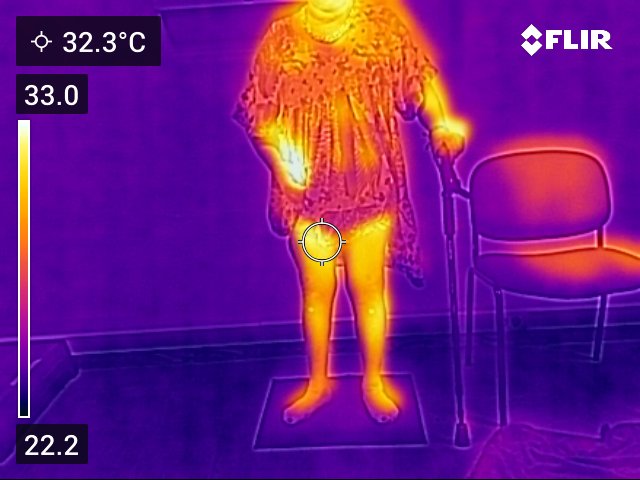

Supplement: Supplemental Information 2 [file peerj-13-19843-s002.zip › Fotos termografía/10_PRE_Anterior.jpg]

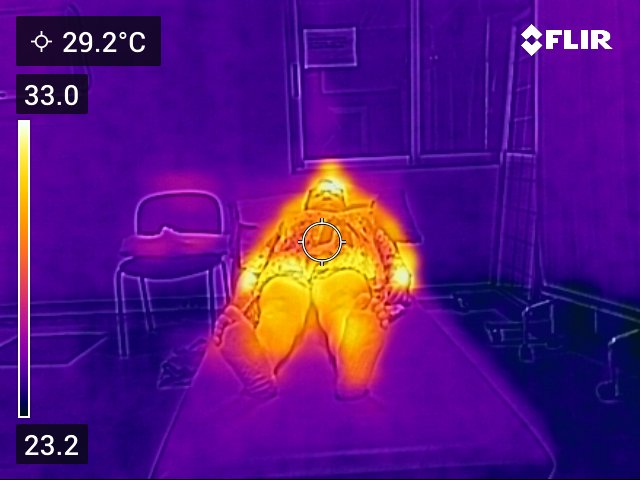

Supplement: Supplemental Information 2 [file peerj-13-19843-s002.zip › Fotos termografía/10_PRE_Pies.jpg]

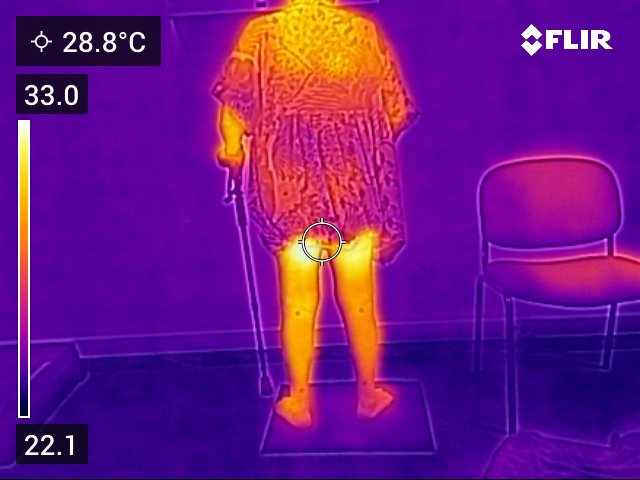

Supplement: Supplemental Information 2 [file peerj-13-19843-s002.zip › Fotos termografía/10_PRE_Posterior.jpg]

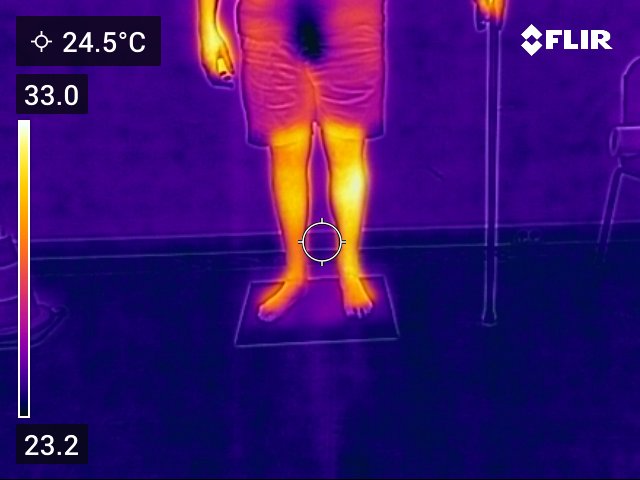

Supplement: Supplemental Information 2 [file peerj-13-19843-s002.zip › Fotos termografía/11_POST_Anterior.jpg]

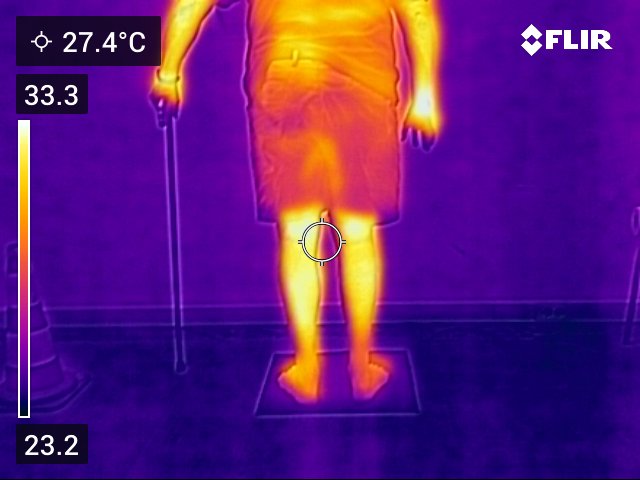

Supplement: Supplemental Information 2 [file peerj-13-19843-s002.zip › Fotos termografía/11_POST_Posterior.jpg]

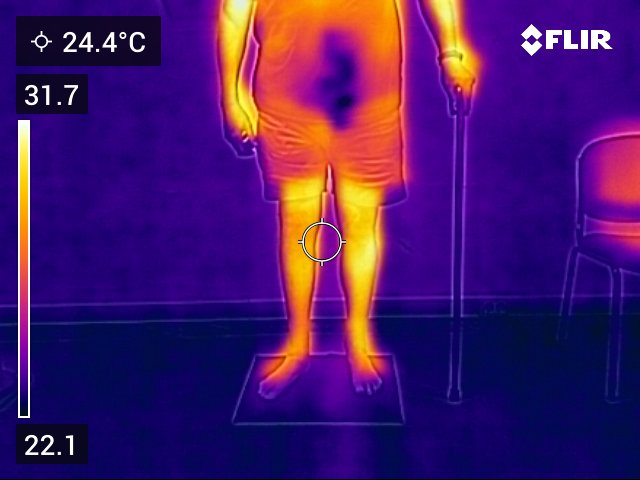

Supplement: Supplemental Information 2 [file peerj-13-19843-s002.zip › Fotos termografía/11_PRE_Anterior.jpg]

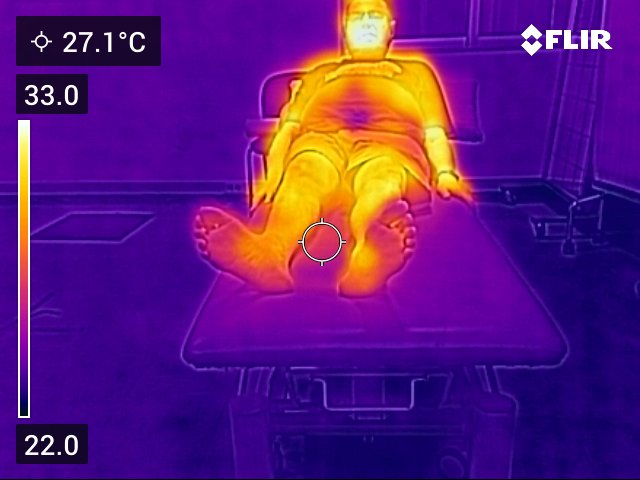

Supplement: Supplemental Information 2 [file peerj-13-19843-s002.zip › Fotos termografía/11_PRE_Pies.jpg]

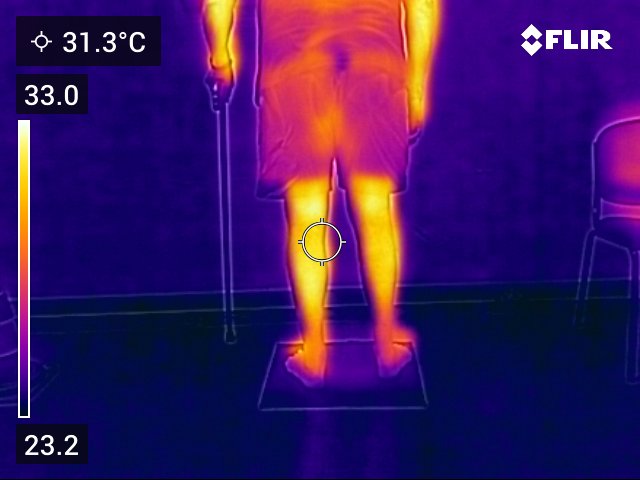

Supplement: Supplemental Information 2 [file peerj-13-19843-s002.zip › Fotos termografía/11_PRE_Posterior.jpg]

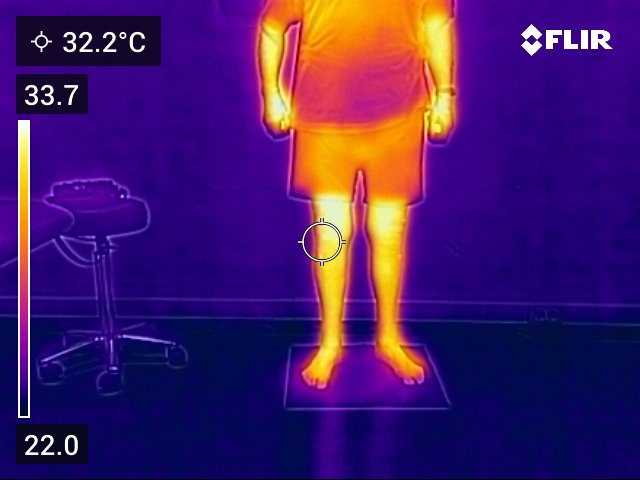

Supplement: Supplemental Information 2 [file peerj-13-19843-s002.zip › Fotos termografía/2_POST_Anterior.jpg]

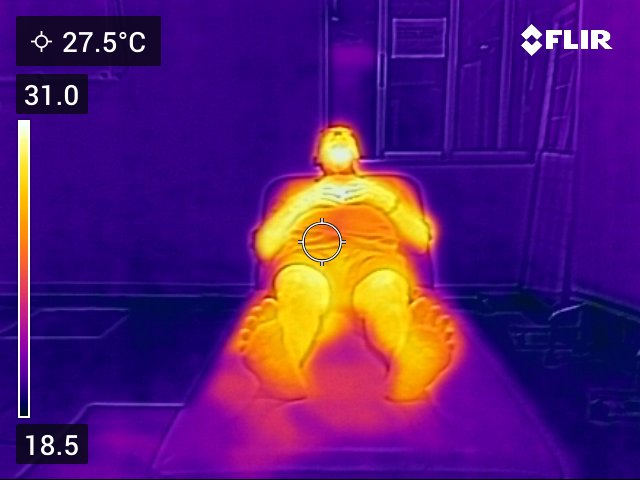

Supplement: Supplemental Information 2 [file peerj-13-19843-s002.zip › Fotos termografía/2_POST_Pies.jpg]

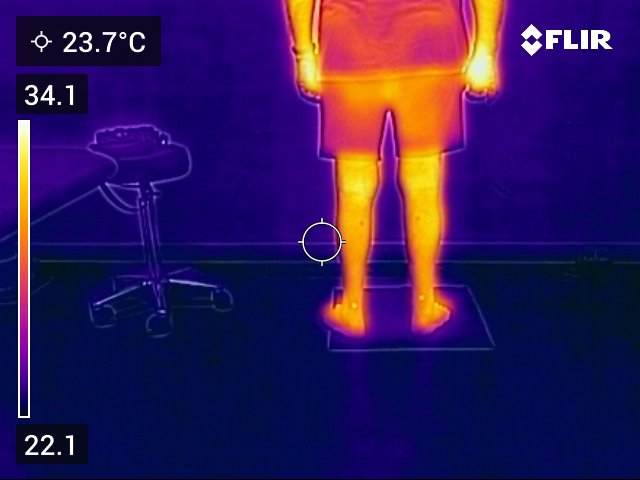

Supplement: Supplemental Information 2 [file peerj-13-19843-s002.zip › Fotos termografía/2_POST_Posterior.jpg]

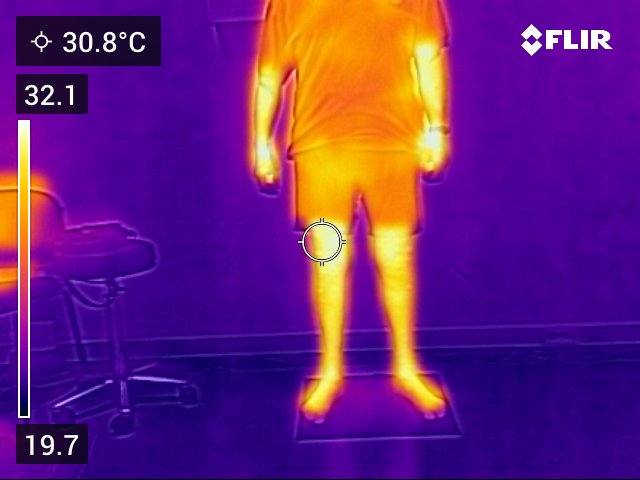

Supplement: Supplemental Information 2 [file peerj-13-19843-s002.zip › Fotos termografía/2_PRE_Anterior.jpg]

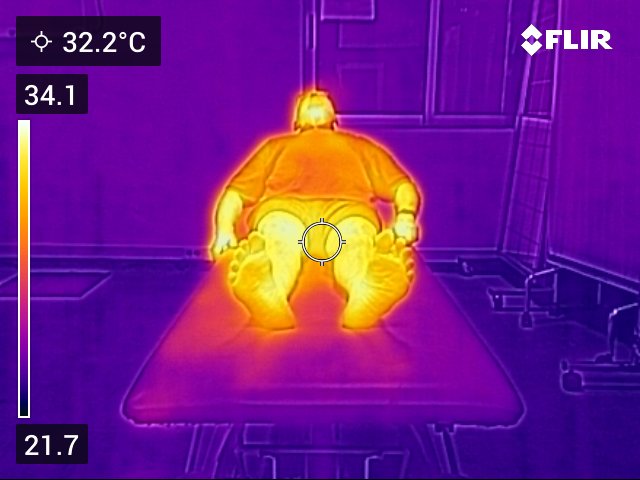

Supplement: Supplemental Information 2 [file peerj-13-19843-s002.zip › Fotos termografía/2_PRE_Pies.jpg]

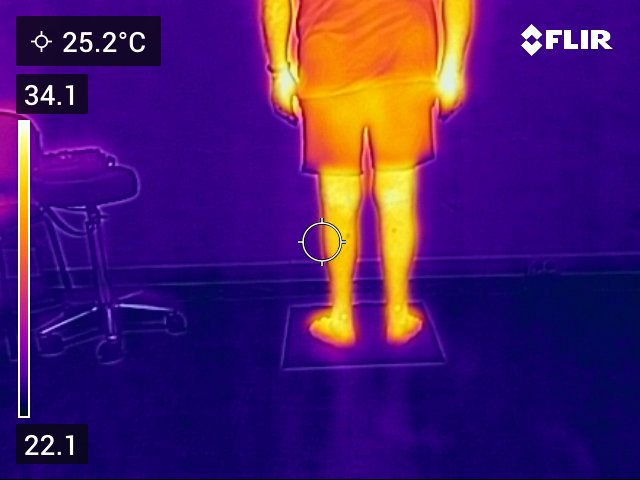

Supplement: Supplemental Information 2 [file peerj-13-19843-s002.zip › Fotos termografía/2_PRE_Posterior.jpg]

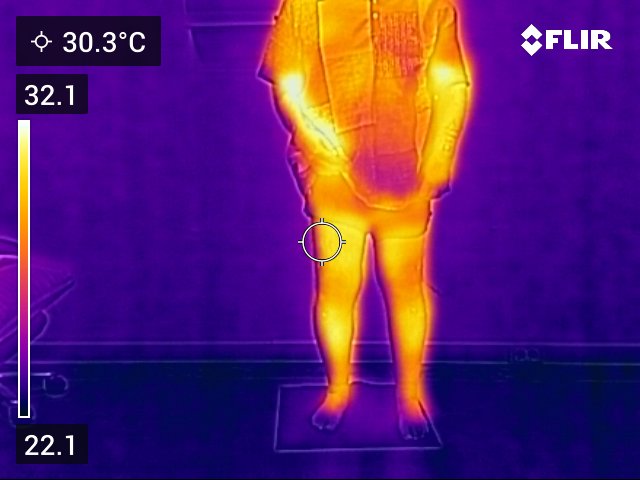

Supplement: Supplemental Information 2 [file peerj-13-19843-s002.zip › Fotos termografía/3_POST_Anterior.jpg]

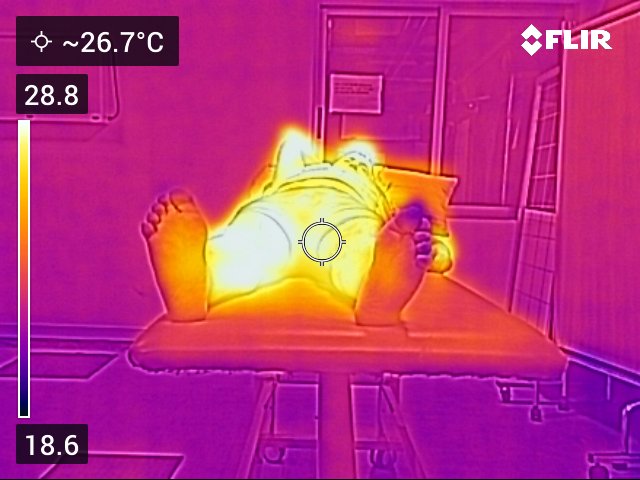

Supplement: Supplemental Information 2 [file peerj-13-19843-s002.zip › Fotos termografía/3_POST_Pies.jpg]

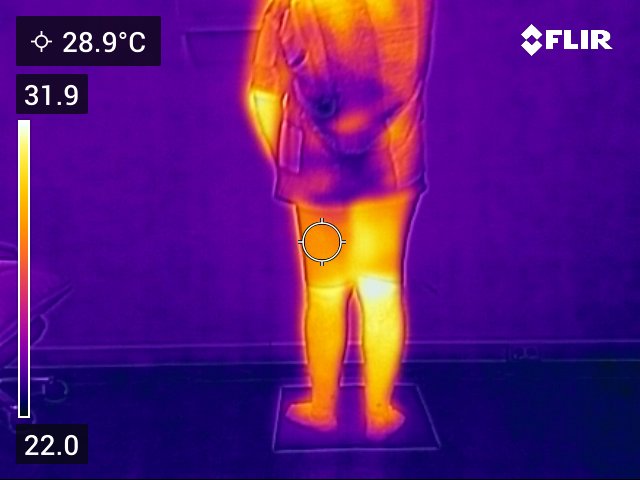

Supplement: Supplemental Information 2 [file peerj-13-19843-s002.zip › Fotos termografía/3_POST_Posterior.jpg]

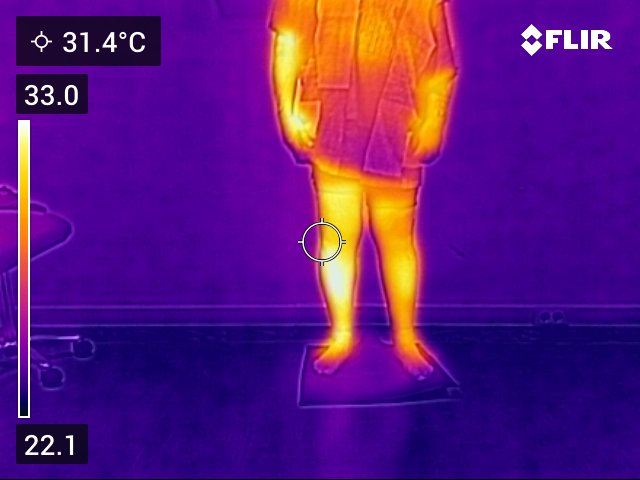

Supplement: Supplemental Information 2 [file peerj-13-19843-s002.zip › Fotos termografía/3_PRE_Anterior.jpg]

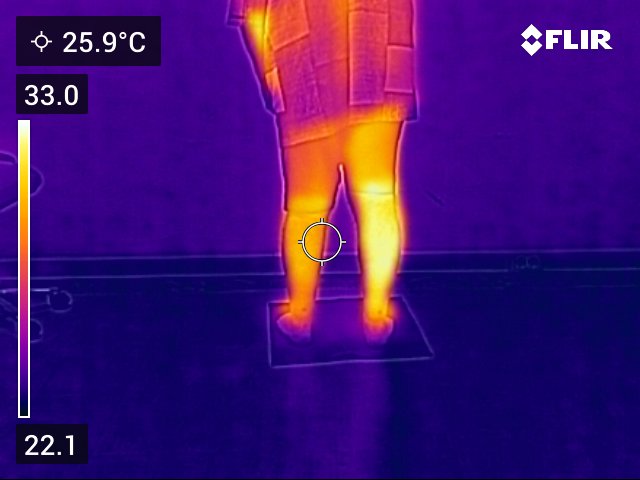

Supplement: Supplemental Information 2 [file peerj-13-19843-s002.zip › Fotos termografía/3_PRE_Posterior.jpg]

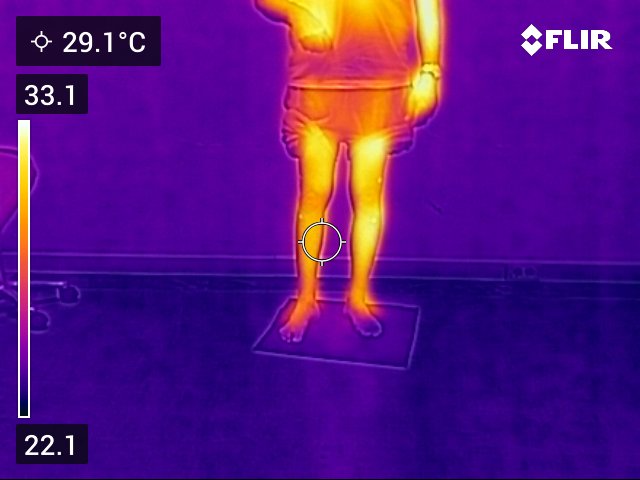

Supplement: Supplemental Information 2 [file peerj-13-19843-s002.zip › Fotos termografía/4_POST_Anterior.jpg]

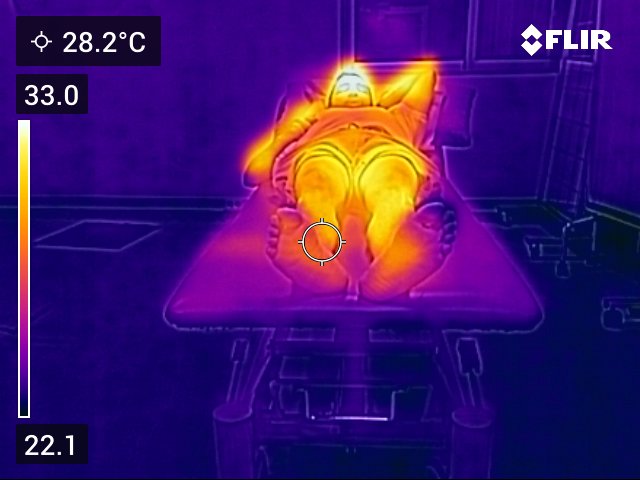

Supplement: Supplemental Information 2 [file peerj-13-19843-s002.zip › Fotos termografía/4_POST_Pies.jpg]

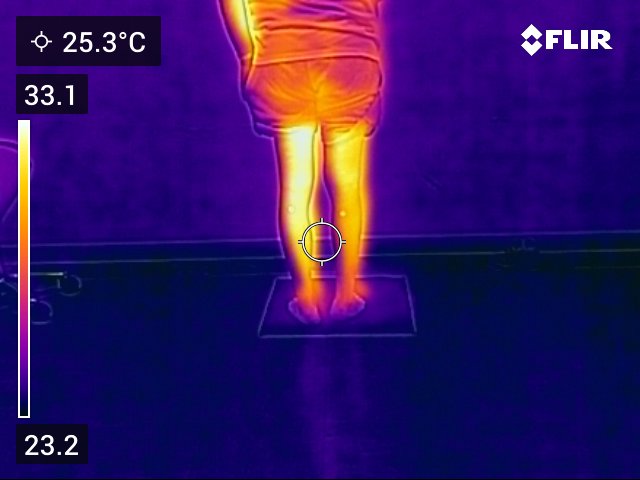

Supplement: Supplemental Information 2 [file peerj-13-19843-s002.zip › Fotos termografía/4_POST_Posterior.jpg]

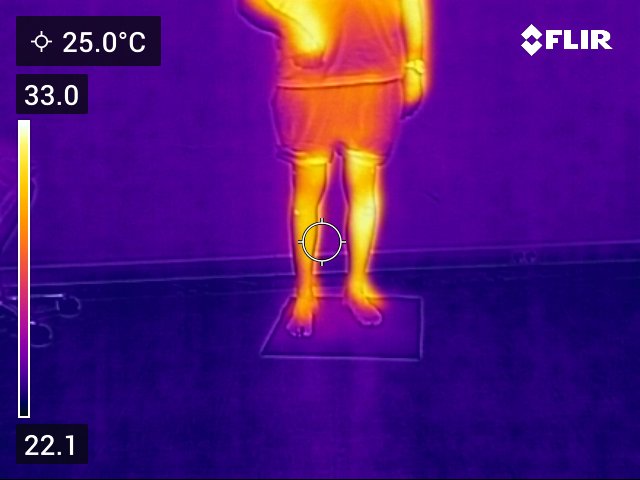

Supplement: Supplemental Information 2 [file peerj-13-19843-s002.zip › Fotos termografía/4_PRE_Anterior.jpg]

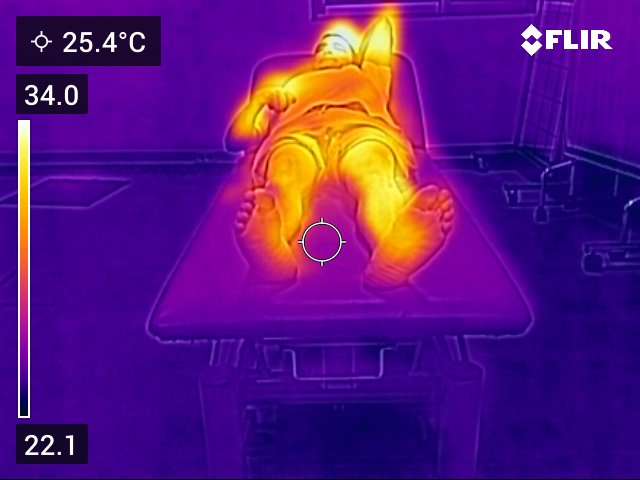

Supplement: Supplemental Information 2 [file peerj-13-19843-s002.zip › Fotos termografía/4_PRE_Pies.jpg]

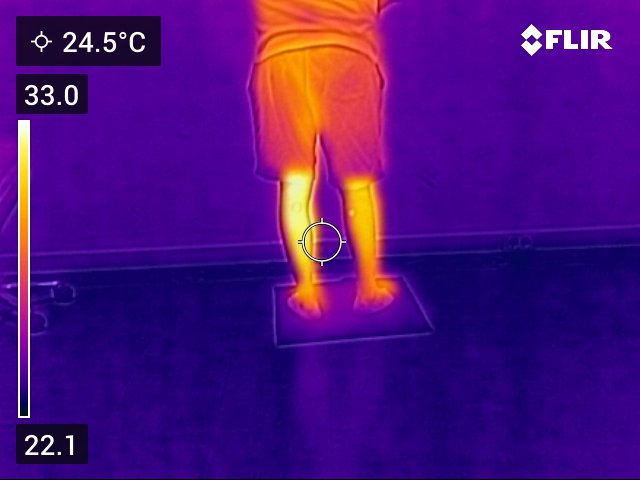

Supplement: Supplemental Information 2 [file peerj-13-19843-s002.zip › Fotos termografía/4_PRE_Posterior.jpg]

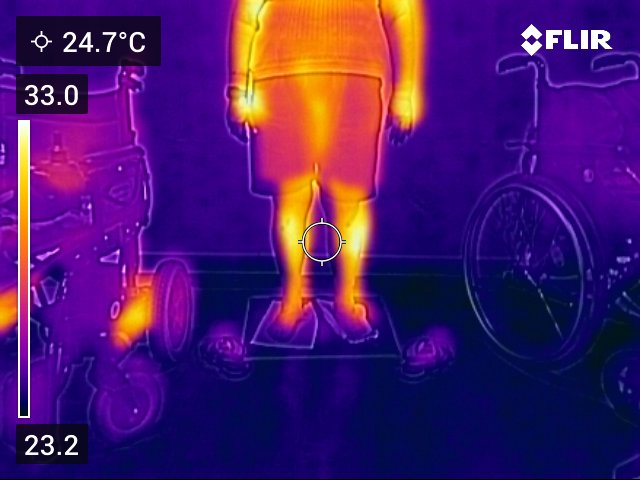

Supplement: Supplemental Information 2 [file peerj-13-19843-s002.zip › Fotos termografía/5_POST_Anterior.jpg]

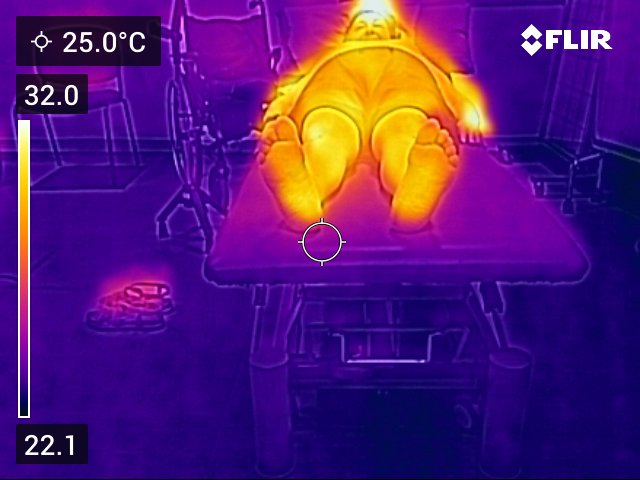

Supplement: Supplemental Information 2 [file peerj-13-19843-s002.zip › Fotos termografía/5_POST_Pies.jpg]

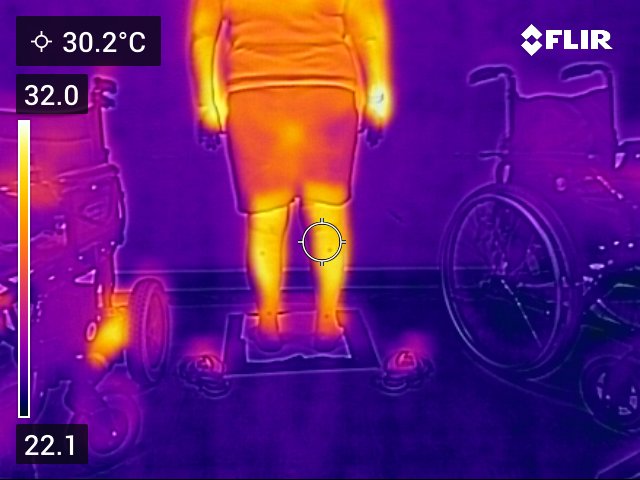

Supplement: Supplemental Information 2 [file peerj-13-19843-s002.zip › Fotos termografía/5_POST_Posterior.jpg]

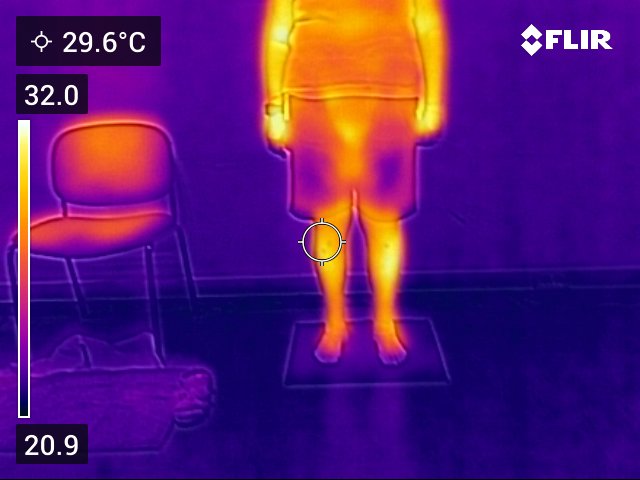

Supplement: Supplemental Information 2 [file peerj-13-19843-s002.zip › Fotos termografía/5_PRE_Anterior.jpg]

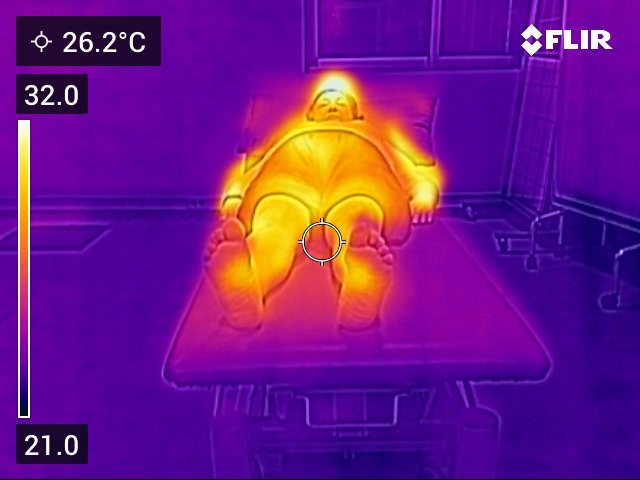

Supplement: Supplemental Information 2 [file peerj-13-19843-s002.zip › Fotos termografía/5_PRE_Pies.jpg]

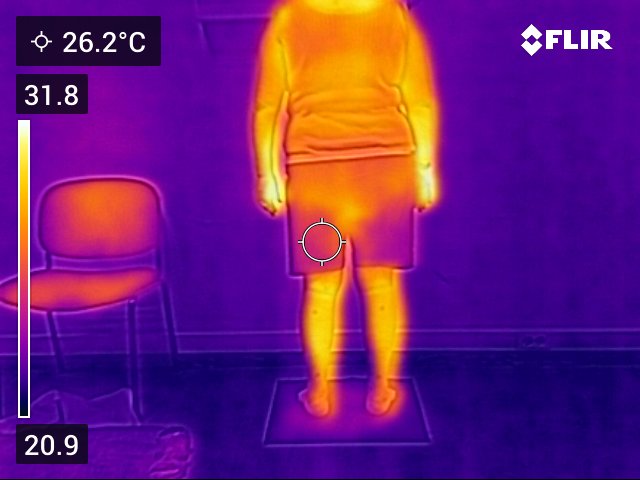

Supplement: Supplemental Information 2 [file peerj-13-19843-s002.zip › Fotos termografía/5_PRE_Posterior.jpg]

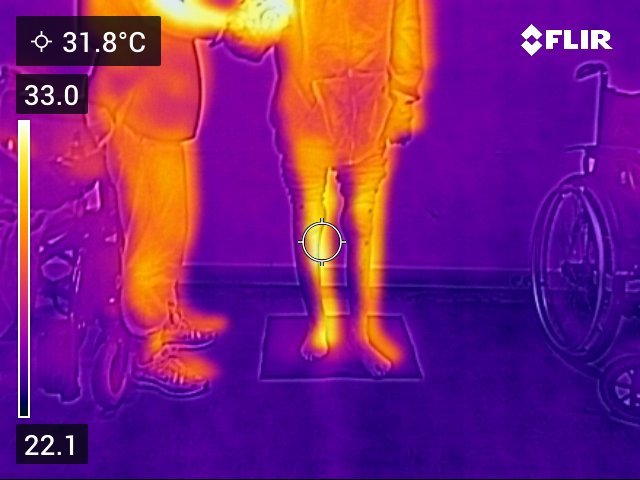

Supplement: Supplemental Information 2 [file peerj-13-19843-s002.zip › Fotos termografía/6_POST_Anterior.jpg]

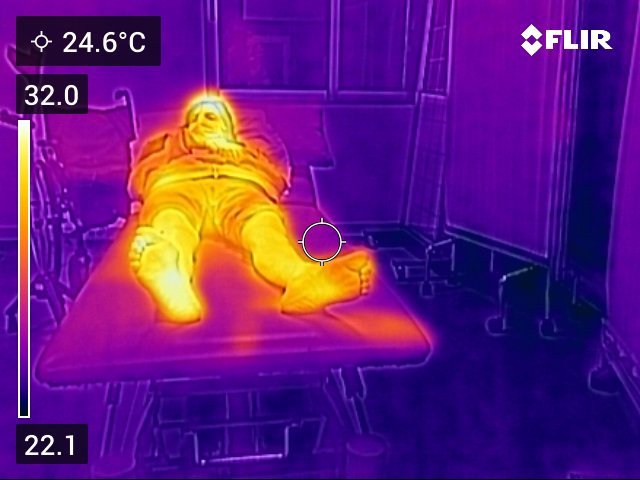

Supplement: Supplemental Information 2 [file peerj-13-19843-s002.zip › Fotos termografía/6_POST_Pies.jpg]

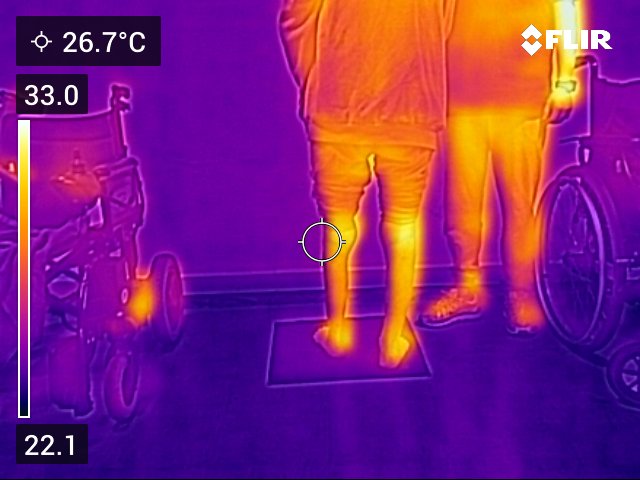

Supplement: Supplemental Information 2 [file peerj-13-19843-s002.zip › Fotos termografía/6_POST_Posterior.jpg]

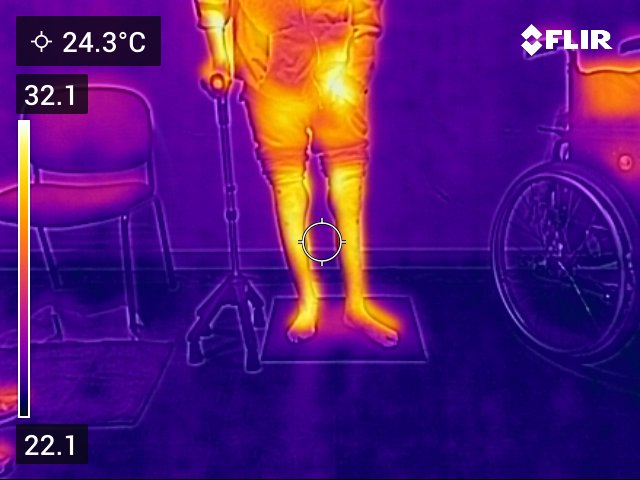

Supplement: Supplemental Information 2 [file peerj-13-19843-s002.zip › Fotos termografía/6_PRE_Anterior.jpg]

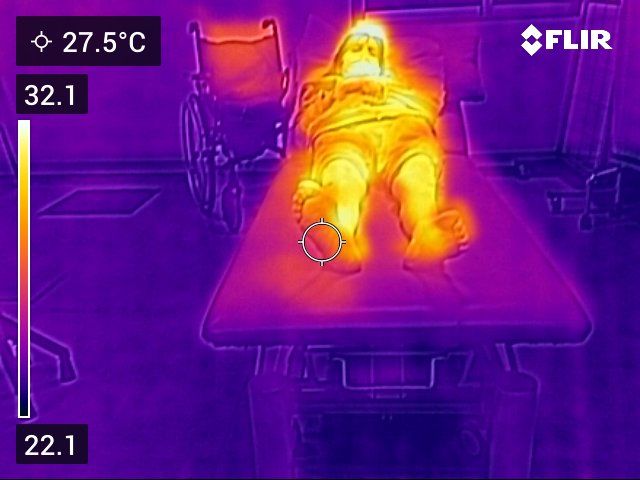

Supplement: Supplemental Information 2 [file peerj-13-19843-s002.zip › Fotos termografía/6_PRE_Pies.jpg]

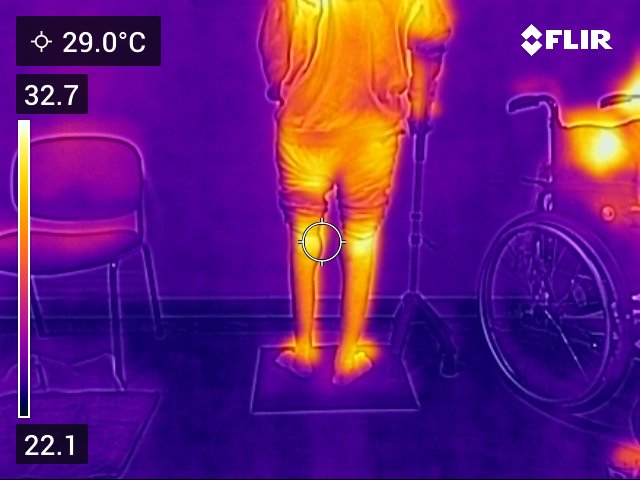

Supplement: Supplemental Information 2 [file peerj-13-19843-s002.zip › Fotos termografía/6_PRE_Posterior.jpg]

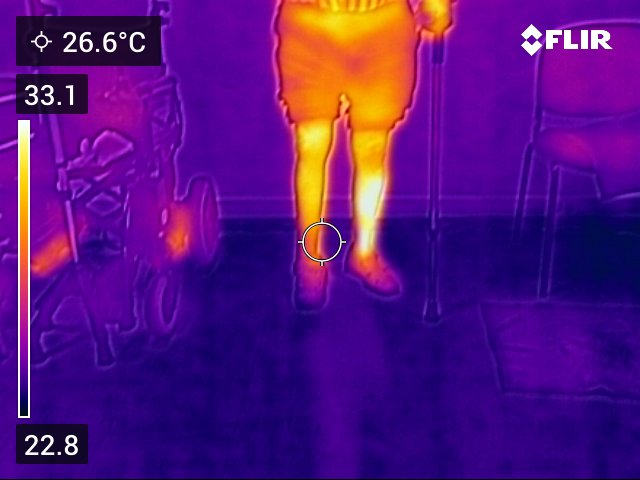

Supplement: Supplemental Information 2 [file peerj-13-19843-s002.zip › Fotos termografía/7_POST_Anterior.jpg]

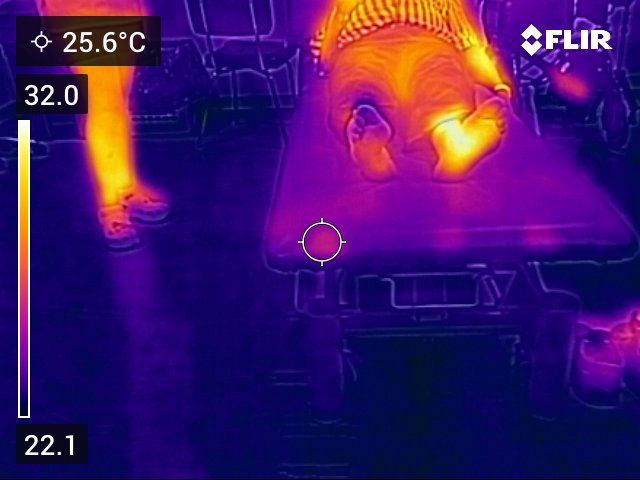

Supplement: Supplemental Information 2 [file peerj-13-19843-s002.zip › Fotos termografía/7_POST_Pies.jpg]

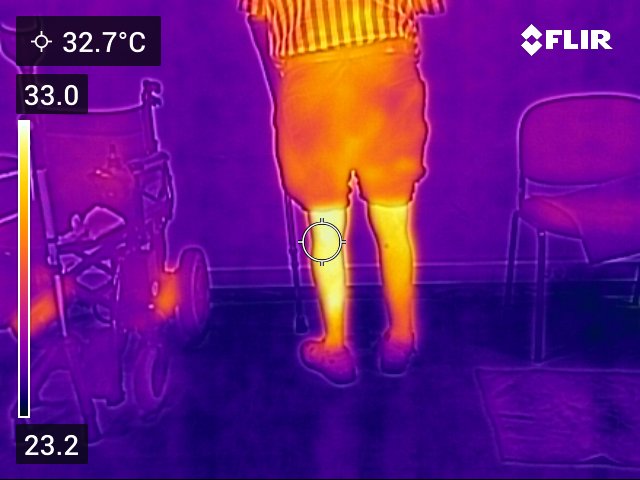

Supplement: Supplemental Information 2 [file peerj-13-19843-s002.zip › Fotos termografía/7_POST_Posterior.jpg]

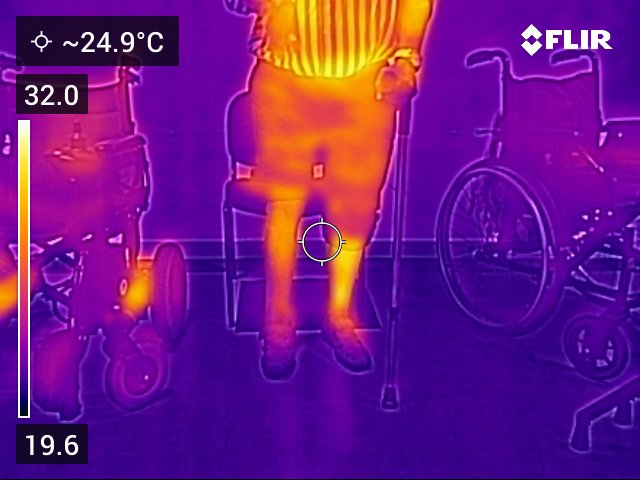

Supplement: Supplemental Information 2 [file peerj-13-19843-s002.zip › Fotos termografía/7_PRE_Anterior.jpg]

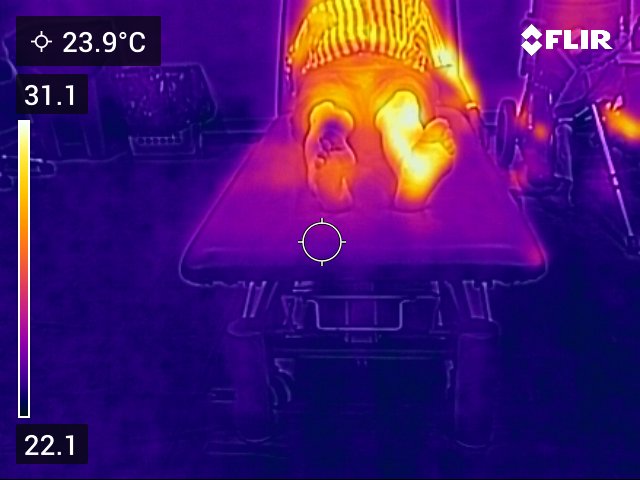

Supplement: Supplemental Information 2 [file peerj-13-19843-s002.zip › Fotos termografía/7_PRE_Pies.jpg]

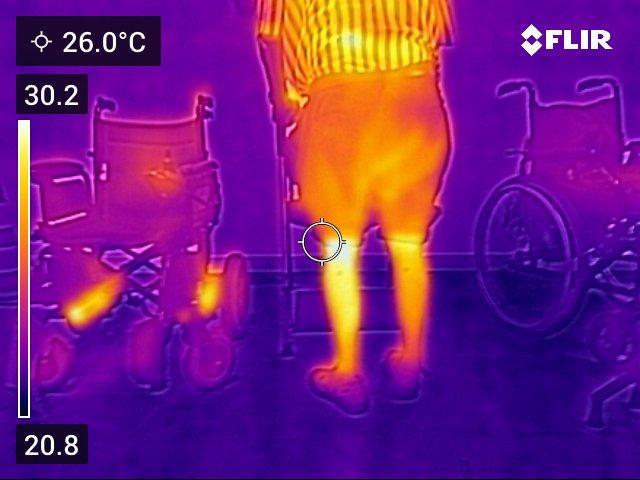

Supplement: Supplemental Information 2 [file peerj-13-19843-s002.zip › Fotos termografía/7_PRE_Posterior.jpg]

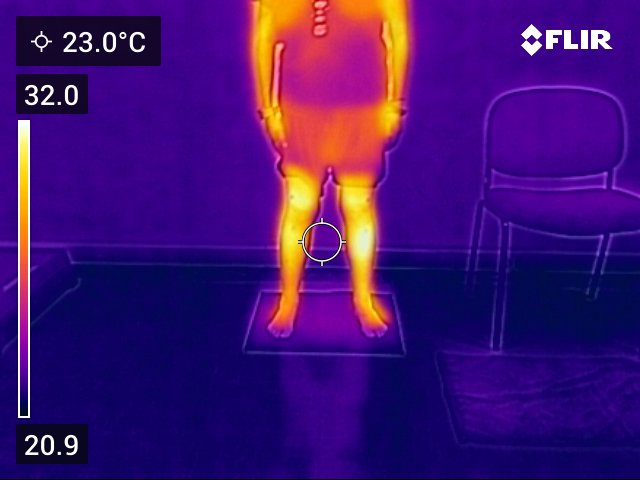

Supplement: Supplemental Information 2 [file peerj-13-19843-s002.zip › Fotos termografía/8_POST_Anterior.jpg]

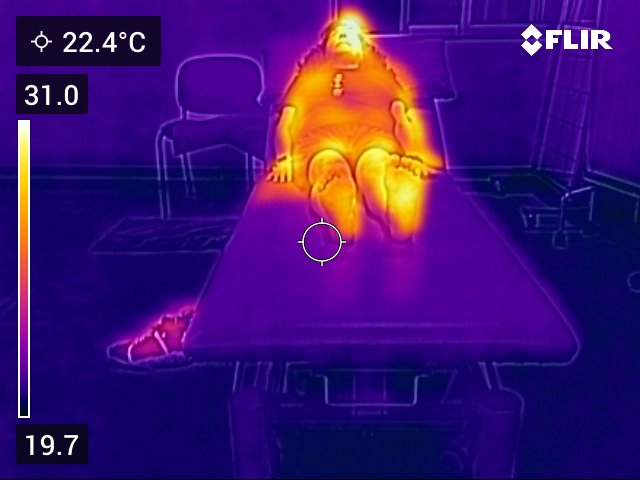

Supplement: Supplemental Information 2 [file peerj-13-19843-s002.zip › Fotos termografía/8_POST_Pies.jpg]

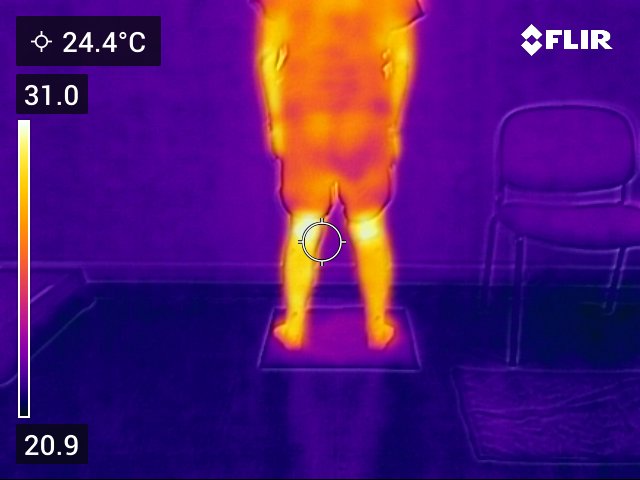

Supplement: Supplemental Information 2 [file peerj-13-19843-s002.zip › Fotos termografía/8_POST_Posterior.jpg]

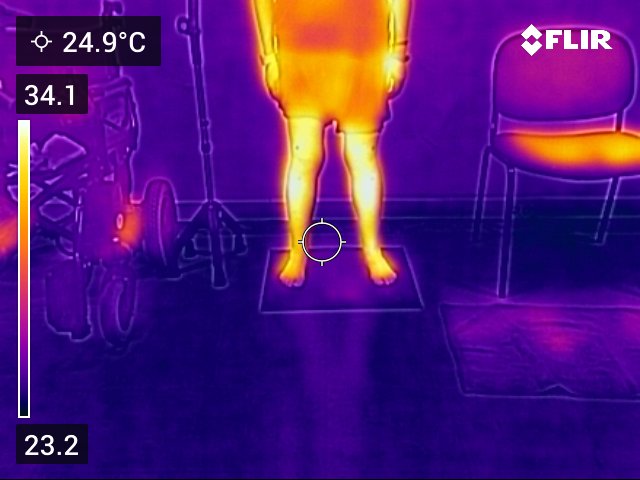

Supplement: Supplemental Information 2 [file peerj-13-19843-s002.zip › Fotos termografía/8_PRE_Anterior.jpg]

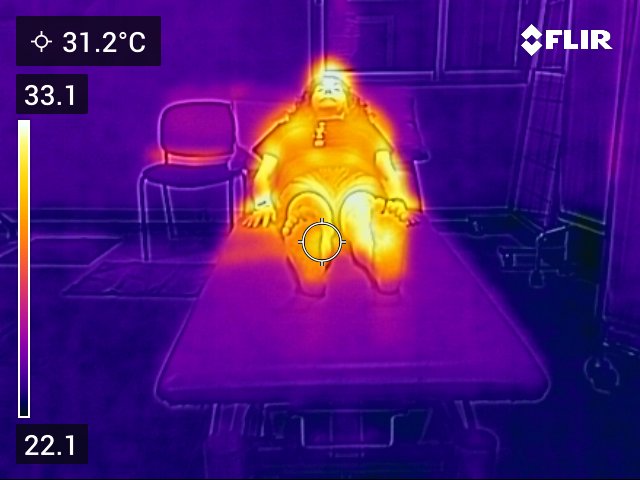

Supplement: Supplemental Information 2 [file peerj-13-19843-s002.zip › Fotos termografía/8_PRE_Pies.jpg]

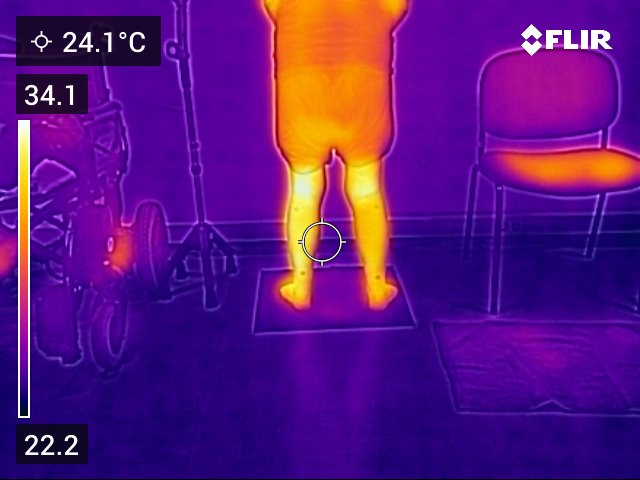

Supplement: Supplemental Information 2 [file peerj-13-19843-s002.zip › Fotos termografía/8_PRE_Posterior.jpg]

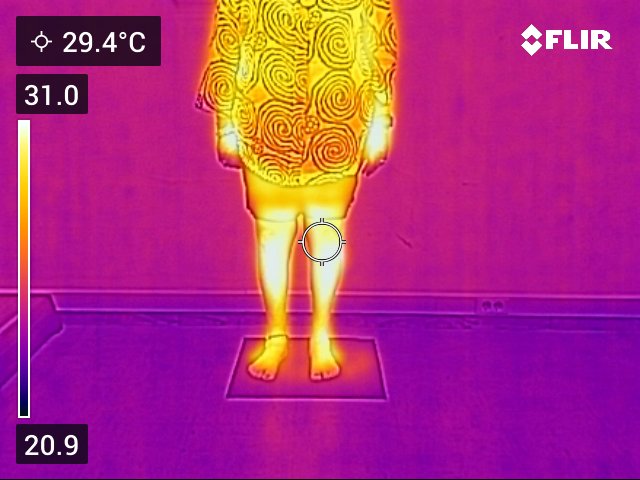

Supplement: Supplemental Information 2 [file peerj-13-19843-s002.zip › Fotos termografía/9_POST_Anterior.jpg]

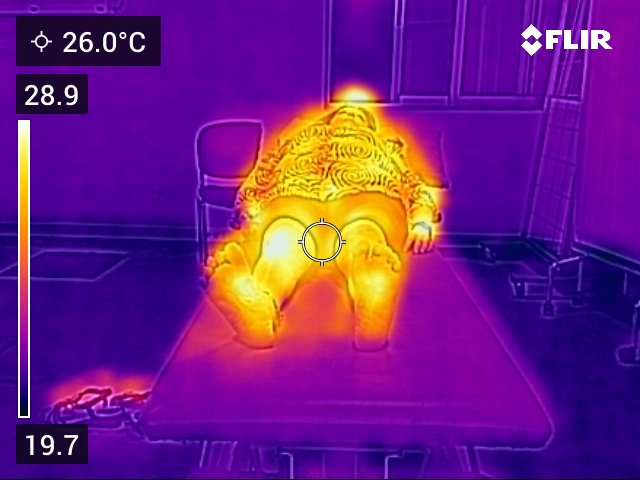

Supplement: Supplemental Information 2 [file peerj-13-19843-s002.zip › Fotos termografía/9_POST_Pies.jpg]

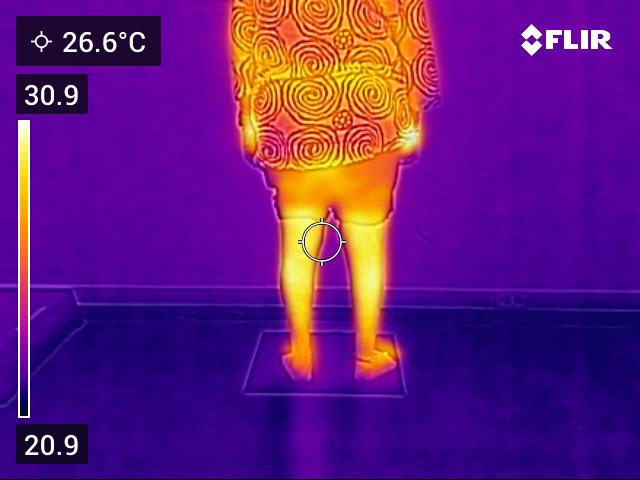

Supplement: Supplemental Information 2 [file peerj-13-19843-s002.zip › Fotos termografía/9_POST_Posterior.jpg]

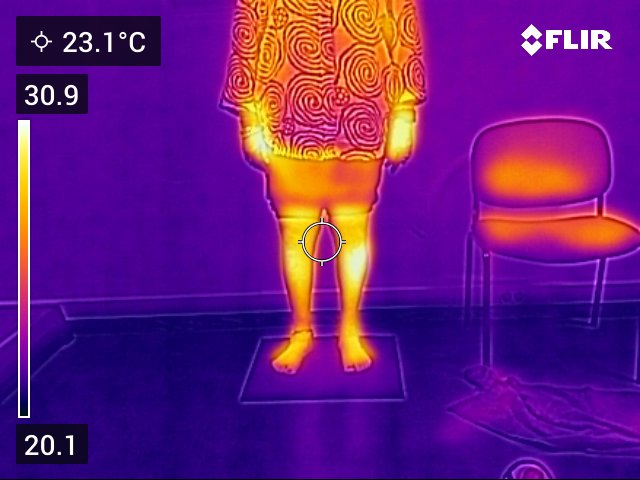

Supplement: Supplemental Information 2 [file peerj-13-19843-s002.zip › Fotos termografía/9_PRE_Anterior.jpg]

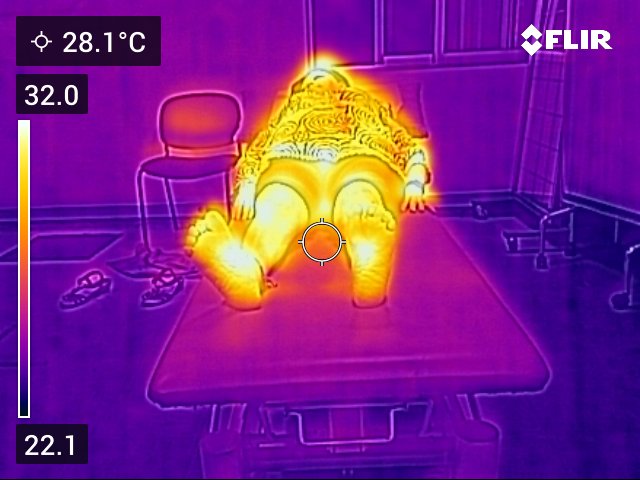

Supplement: Supplemental Information 2 [file peerj-13-19843-s002.zip › Fotos termografía/9_PRE_Pies.jpg]

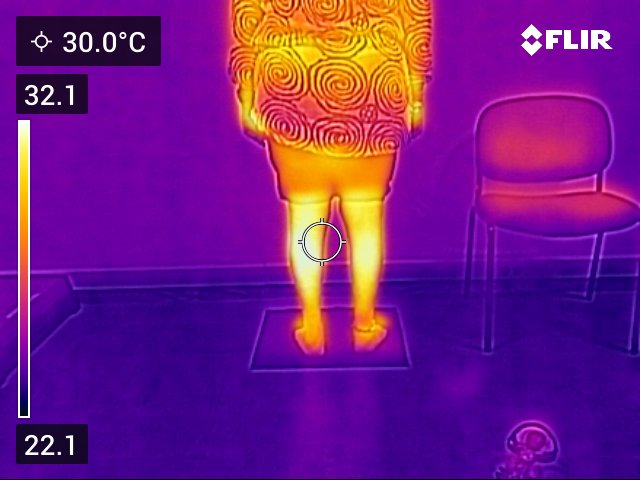

Supplement: Supplemental Information 2 [file peerj-13-19843-s002.zip › Fotos termografía/9_PRE_Posterior.jpg]

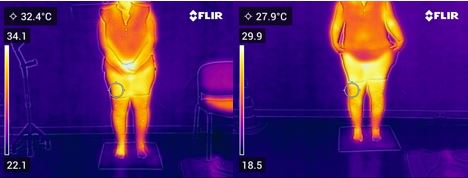

Supplement: Supplemental Information 2 [file peerj-13-19843-s002.zip › Fotos termografía/CARA ANTERIOR 1.JPG]

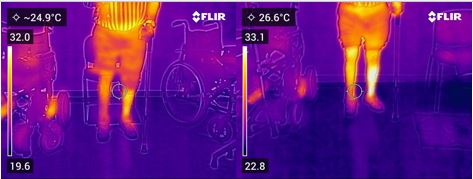

Supplement: Supplemental Information 2 [file peerj-13-19843-s002.zip › Fotos termografía/CARA ANTERIOR 7.JPG]

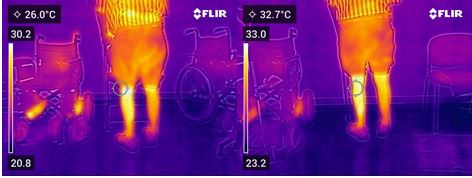

Supplement: Supplemental Information 2 [file peerj-13-19843-s002.zip › Fotos termografía/CARA POSTERIOR 7.JPG]

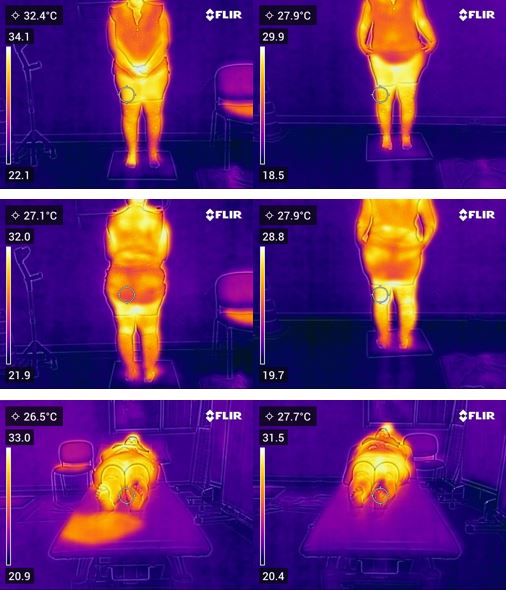

Supplement: Supplemental Information 2 [file peerj-13-19843-s002.zip › Fotos termografía/PACIENTE 1.JPG]

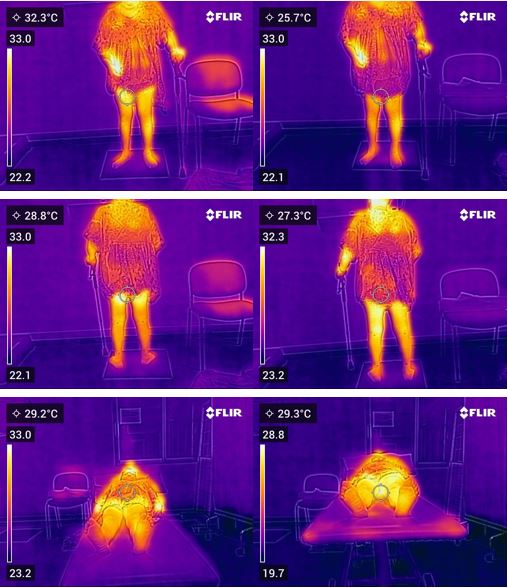

Supplement: Supplemental Information 2 [file peerj-13-19843-s002.zip › Fotos termografía/PACIENTE 10.JPG]

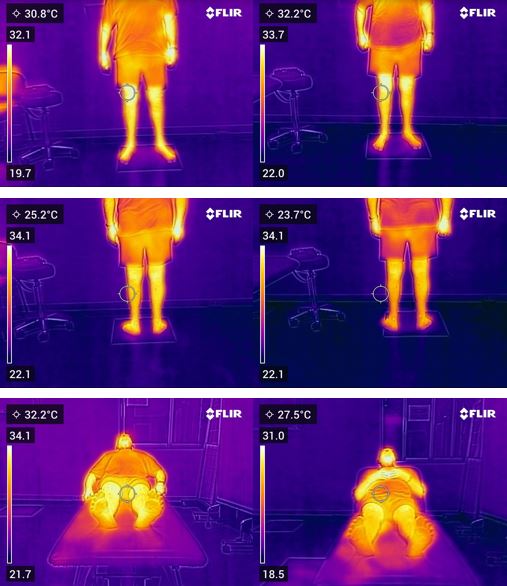

Supplement: Supplemental Information 2 [file peerj-13-19843-s002.zip › Fotos termografía/PACIENTE 2.JPG]

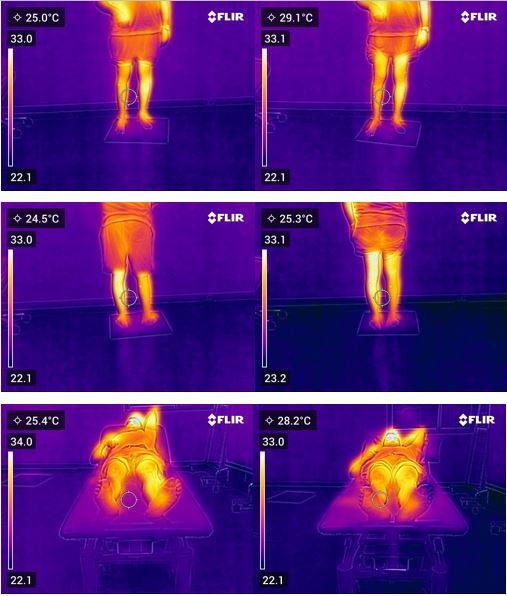

Supplement: Supplemental Information 2 [file peerj-13-19843-s002.zip › Fotos termografía/PACIENTE 4.JPG]

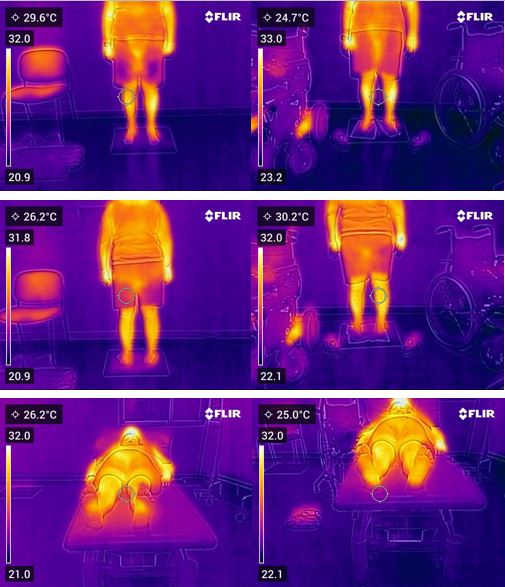

Supplement: Supplemental Information 2 [file peerj-13-19843-s002.zip › Fotos termografía/PACIENTE 5.JPG]

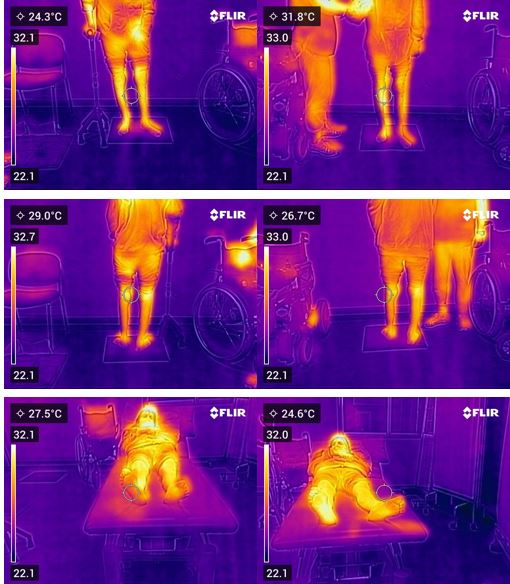

Supplement: Supplemental Information 2 [file peerj-13-19843-s002.zip › Fotos termografía/PACIENTE 6.JPG]

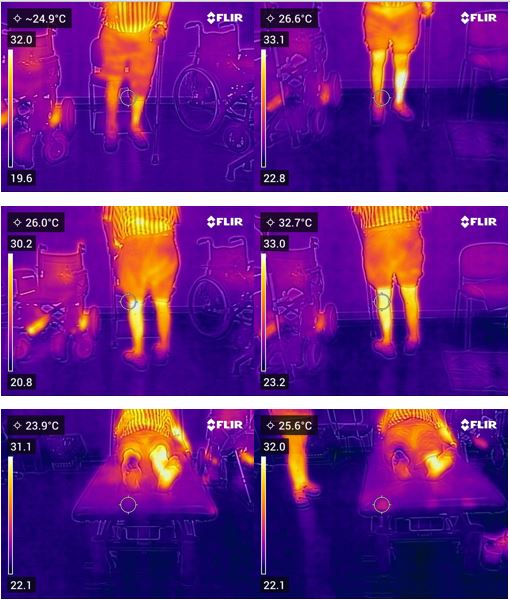

Supplement: Supplemental Information 2 [file peerj-13-19843-s002.zip › Fotos termografía/PACIENTE 7.JPG]

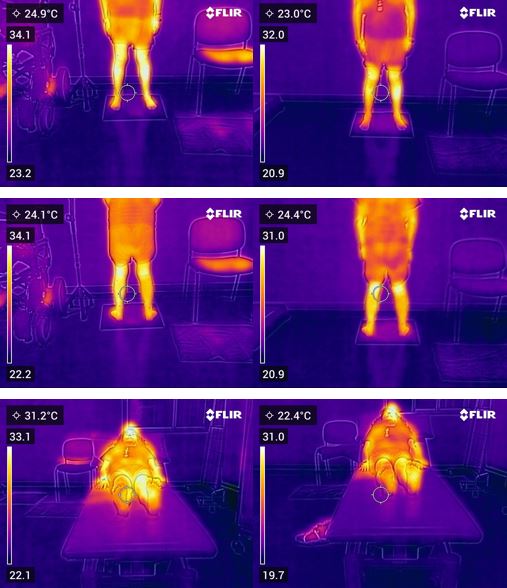

Supplement: Supplemental Information 2 [file peerj-13-19843-s002.zip › Fotos termografía/PACIENTE 8.JPG]

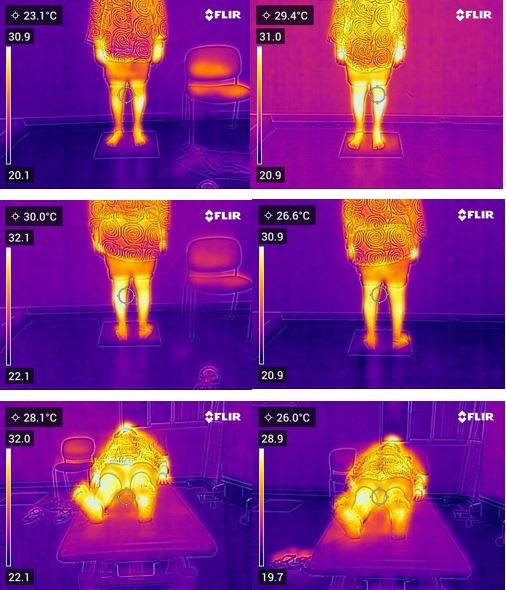

Supplement: Supplemental Information 2 [file peerj-13-19843-s002.zip › Fotos termografía/PACIENTE 9.JPG]

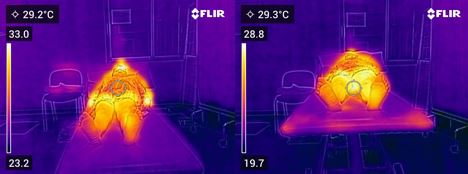

Supplement: Supplemental Information 2 [file peerj-13-19843-s002.zip › Fotos termografía/PIES 10.JPG]

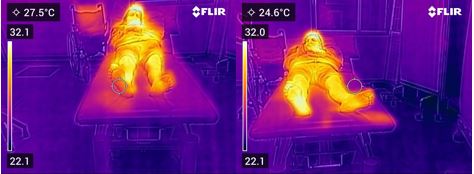

Supplement: Supplemental Information 2 [file peerj-13-19843-s002.zip › Fotos termografía/PIES 6.JPG]
