# Supplementary material for: Thermography analysis as a tool for assessing thermal asymmetries and temperature changes after therapy in patients with stroke: a pilot study
Source: PeerJ. 2025 Aug 28;13:e19843. doi: 10.7717/peerj.19843 (PMC12399083; doi:10.7717/peerj.19843)
Supplement: Supplemental Information 3 [file peerj-13-19843-s003.doc]

STROBE Statement—Checklist of items that should be included in reports of ***cohort studies***

|  | Item No | Recommendation |
| --- | --- | --- |
| **Title and abstract** | 1 | (*a*) Indicate the study’s design with a commonly used term in the title or the abstract  Quasi-experimental pilot study as stated in the Abstract page 1 and Methodology page 2. |
|  |  | (*b*) Provide in the abstract an informative and balanced summary of what was done and what was found  Provide in Abstract at page 1 |
| Introduction | | |
| Background/rationale | 2 | Explain the scientific background and rationale for the investigation being reported  Included in Introduction page 1-2 |
| Objectives | 3 | State specific objectives, including any prespecified hypotheses  Included in Introduction page 1-2 |
| Methods | | |
| Study design | 4 | Present key elements of study design early in the paper  Included in the Methodology page 2,3 and 4 |
| Setting | 5 | Describe the setting, locations, and relevant dates, including periods of recruitment, exposure, follow-up, and data collection  Included in the Methodology- Procedure page 3 |
| Participants | 6 | (*a*) Give the eligibility criteria, and the sources and methods of selection of participants. Describe methods of follow-up  Included in the Methodology- Participants page 2-3 |
|  |  | (*b*) For matched studies, give matching criteria and number of exposed and unexposed  *N/A* |
| Variables | 7 | Clearly define all outcomes, exposures, predictors, potential confounders, and effect modifiers. Give diagnostic criteria, if applicable  Included in the Methodology- Procedure at page 3-4.. |
| Data sources/ measurement | 8* | For each variable of interest, give sources of data and details of methods of assessment (measurement). Describe comparability of assessment methods if there is more than one group  Included in the Methodology- Procedure at page 3-4. |
| Bias | 9 | Describe any efforts to address potential sources of bias  Included in the Methodology- Procedure at page 3-4. |
| Study size | 10 | Explain how the study size was arrived at  Included in the Methodology- Participants at page 2-3 |
| Quantitative variables | 11 | Explain how quantitative variables were handled in the analyses. If applicable, describe which groupings were chosen and why  Included in the Methodology- Procedure at page 4-5. |
| Statistical methods | 12 | (*a*) Describe all statistical methods, including those used to control for confounding  Included in the Methodology- Statistical analysis at page 5. |
|  |  | (*b*) Describe any methods used to examine subgroups and interactions  Included in the Methodology- Statistical analysis at page 5 |
|  |  | (*c*) Explain how missing data were addressed  N/A |
|  |  | (*d*) If applicable, explain how loss to follow-up was addressed  N/A |
|  |  | (*e*) Describe any sensitivity analyses  N/A |
| Results | | |
| Participants | 13* | (a) Report numbers of individuals at each stage of study—eg numbers potentially eligible, examined for eligibility, confirmed eligible, included in the study, completing follow-up, and analysed  N/A |
|  |  | (b) Give reasons for non-participation at each stage  N/A |
|  |  | (c) Consider use of a flow diagram  N/A |
| Descriptive data | 14* | (a) Give characteristics of study participants (eg demographic, clinical, social) and information on exposures and potential confounders  Included in the Results section at page 5 |
|  |  | (b) Indicate number of participants with missing data for each variable of interest  N/A. |
|  |  | (c) Summarise follow-up time (eg, average and total amount)  N/A. |
| Outcome data | 15* | Report numbers of outcome events or summary measures over time  Lines |
| Main results | 16 | (*a*) Give unadjusted estimates and, if applicable, confounder-adjusted estimates and their precision (eg, 95% confidence interval). Make clear which confounders were adjusted for and why they were included  Included in the Results section at page 6-7 |
|  |  | (*b*) Report category boundaries when continuous variables were categorized  Included in the Results section at page 7 |
|  |  | (*c*) If relevant, consider translating estimates of relative risk into absolute risk for a meaningful time period N/A |
| Other analyses | 17 | Report other analyses done—eg analyses of subgroups and interactions, and sensitivity analyses  N/A |
| Discussion | | |
| Key results | 18 | Summarise key results with reference to study objectives  Included in the Discussion section at page 7,8 and 9 |
| Limitations | 19 | Discuss limitations of the study, taking into account sources of potential bias or imprecision. Discuss both direction and magnitude of any potential bias  Included in the Limitations section at page 9 |
| Interpretation | 20 | Give a cautious overall interpretation of results considering objectives, limitations, multiplicity of analyses, results from similar studies, and other relevant evidence  Included in the Conclusion section page 9 |
| Generalisability | 21 | Discuss the generalisability (external validity) of the study results  Included in the Limitations and Conclusion sections at page 9 |
| Other information | | |
| Funding | 22 | Give the source of funding and the role of the funders for the present study and, if applicable, for the original study on which the present article is based  Included in the Declarations at page 10 |

*Give information separately for exposed and unexposed groups.

**Note:** An Explanation and Elaboration article discusses each checklist item and gives methodological background and published examples of transparent reporting. The STROBE checklist is best used in conjunction with this article (freely available on the Web sites of PLoS Medicine at http://www.plosmedicine.org/, Annals of Internal Medicine at http://www.annals.org/, and Epidemiology at http://www.epidem.com/). Information on the STROBE Initiative is available at http://www.strobe-statement.org.
